# Antibiotic resistance profiles in cultivable microbiota isolated from some romanian natural fishery lakes included in Natura 2000 network

**DOI:** 10.1186/s12917-021-02770-8

**Published:** 2021-01-26

**Authors:** Veronica Lazăr, Irina Gheorghe, Carmen Curutiu, Ioana Savin, Florica Marinescu, Violeta Corina Cristea, Dumitru Dobre, Gabriela Loredana Popa, Mariana Carmen Chifiriuc, Mircea Ioan Popa

**Affiliations:** 1 Department of Microbiology and Immunology, Faculty of Biology, University of Bucharest, Bucharest, Romania; 2 Research Institute of the University of Bucharest (ICUB), Bucharest, Romania; 3 Maximilian Association, Buzău, Romania; 4 National Institute for Research and Development in Environmental Protection , Bucharest, Romania; 5 Carol Davila University of Medicine and Pharmacy, Bucharest, Romania; 6 Cantacuzino National Medico-Military Institute for Research and Development, Bucharest, Romania; 7 Academy of Romanian Scientists, Bucharest, Romania

**Keywords:** Lowland fishery lakes, Antimicrobial resistance, *Enterococcus* sp., *Enterobacterales*

## Abstract

**Background:**

The present study aims the characterization of antibiotic resistance phenotypes and encoding genes in bacterial strains isolated from some Romanian aquatic fishery lowland salted lakes.

**Material/Methods:**

This study was conducted on 44 bacterial strains, mainly belonging to species used as microbiological indicators of fecal pollution isolated from four natural fishery lakes. All strains were tested for their antibiotic susceptibility by disk diffusion method. Simplex and multiplex PCR were performed to identify the β-lactams antibiotic resistance genes (*bla*_NMD_, *bla*_OXA−48_, *bla*_VIM_, *bla*_IMP_, *bla*_CTX−M_, *bla*_TEM_), sulfonamides (Sul1, Sul2), tetracyclines (TetA, TetB, TetC, TetD, TetM), aminoglycosides (aac3Ia), vancomycin (VanA, VanB, VanC), macrolides (ermA, ermB, ermC) as well as the plasmid-mediated quinolone resistance (PMQR) markers (QnrA, QnrB, QnrS), and class 1 integrons (Int1, drfA1-aadA1).

**Results:**

The *Enterococcus* spp. isolates exhibited phenotypic resistance to vancomycin (35 %) and macrolides (erythromycin) (75 %); from the vancomycin – resistant strains, 5 % harboured VanA (*E. faecalis*), while the erythromycin resistant isolates were positive for the ermA gene (*E. faecalis* − 10 %, *E. faecium* − 5 %). The Gram- negative rods (GNR) exhibited a high level of resistance to β-lactams: cefuroxime (63 %), cefazolin (42 %), ceftriaxone (8 %), ceftazidime and aztreonam (4 % each). The genetic determinants for beta-lactam resistance were represented by *bla*_CTX−M−like_ (33 %), *bla*_NDM−like_ and *bla*_IMP−like_ (8.33 %) genes. The resistance to non-β-lactam antibiotics was ascertained to the following genes: quinolones (QnrS − 4.16 %); sulfonamides (Sul1–75 %, Sul2–4.16 %); aminoglycosides (aac3Ia − 4.16 %); tetracyclines (tetA – 25 %, tetC − 15 %). The integrase gene was found in more than 50 % of the studied strains (58.33 %).

**Conclusions:**

The cultivable aquatic microbiota from fishery lakes is dominated by enterococci and *Enterobacterales* strains. The GNR strains exhibited high levels of β-lactam resistance mediated by extended spectrum beta-lactamases and metallo-β-lactamases. The *Enterococcus* sp. isolates were highly resistant to macrolides and vancomycin. The high level and diversity of resistance markers, correlated with a high frequency of integrons is suggesting that this environment could act as an important reservoir of antibiotic resistance genes with a great probability to be horizontally transmitted to other associated species from the aquatic sediments microbiota, raising the potential zoonotic risk for fish consumers.

## Background

Antimicrobial resistance (AR) is an increasing worldwide concern, as antibiotics still represent a very important option for human and animal health protection [1]. It is expected that the mortality rates due to AR will reach as many as 10 million people/year by 2050, if appropriate action is not taken [2]. AR is now considered without any doubt a typical One Health problem, the environment having a crucial role as a reservoir and transmission route to humans. Consequently, one of the main priorities of the Joint Programme Initiative on Antimicrobial Resistance (JPIAMR) is to foster the research of environmental AR reservoirs, which are very poorly known, in comparison with clinical ones [3–6]. In 2014, Romania reported for the first time to ESVAC the consumption of veterinary antimicrobial agents [7]. At the top of the most-solded classes were tetracyclines, penicillins and aminoglycosides. Similarly, in 2016, the most consumed classes for food-producing animals in Romania according ESVAC were tetracyclines, penicillins, macrolides and aminoglycosides [8]. As antibiotics are one of the most popular pharmaceuticals used in medicine, veterinary care, and farming [9, 10], they can be released, most of them unchanged, into the environment. They appear as contaminants of the water bodies, especially those frequently impacted by anthropogenic activities (e.g., wastewater, municipal sewage, influents and effluents of wastewater treatment plants) [11], which have been suggested to be ideal reservoirs and vectors for the AR origin, evolution and spread [12–14]. In wastewater effluents, lakes, rivers or streams, bacteria from different sources, probably selected by intensive antibiotic usage, are collected and mixed with environmental strains, which on their turn, could introduce the newly acquired antibiotic resistance genes (ARGs) into the clinics [15–17].

Enterococci and *Enterobacterales* species were found in high concentrations in human and animal faeces, so their presence in any type of waters indicate the faecal contamination of the respective water body. The presence of *Enterobacterales* (which do not survive in the water for a long time) indicates a recent faecal water pollution with potentially pathogenic microorganisms originating from anthropogenic systems (household wastewater, livestock), but also with allochtonous species originating from natural ecosystems; enterococci can survive longer in water, due to their high resistance to physical, chemical and biological agents, indicating a chronic faecal contamination of the water [18].

These commensal intestinal bacteria could often act as reservoirs for AR determinants [19]. Therefore, the evaluation of AR profiles of these microbiological indicators of water quality could predict for the ARGs disemination from humans and animals into the environment and the risk of horizontal gene transfer (HGT) to native species.

On the other hand, AR has been detected in different aquatic environments and some resistance determinants, such as the recently emerging plasmid-mediated quinolone resistance determinants (PMQR) from the Qnr family [20] and CTX-M [21] from aquatic *Kluyvera* sp. have been found to originate from aquatic bacteria.

In aquaculture, antibiotics are given as a component of their food, and occasionally in baths and injections [22]. The unconsumed food, as well as the fish faeces, containing antibiotics reach the sediment and the antibiotics could be ingested by wild fish and other organisms, consequently altering the composition of the acquatic microbiota, selecting antibiotic-resistant bacteria (ARB) and favoring the HGT of ARGs [22, 23]. In this study we have investigated the Balta Albă-Amara-Jirlău-Lacul Sărat Câineni lakes, whose vulnerability resides in the spillage of household waste, significant floods and consecutive natural (Buzău river’s flood) and artificial (due to fish farming activities) changes of water composition [24, 25]. These aquatic environments were scarcely studied, the few previous studies focusing on the study of halophilic microorganisms (bacteria and arhaea), phytoplankton and zooplankton species, as well as on the organic compounds hydrolythic activity of the acquatic microbiota isolated from Amara, Balta Albă, Movila Miresei salted lakes and from Ocnele Mari area, in relation with the physical-chemical parameters (pH, density and chloride content) of the respective waters [26, 27]. Our group has previously evaluated the physico-chemical and microbiological characteristics of Balta Albă-Amara-Jirlău-Lacul Sărat Câineni site included in Natura 2000 Network for establishing the degree of organic pollution and generating the knowledge required for the design and implementation of appropriate measures for maintaining the balance between the water protection and the sustainable use of these protected ecosystems [28]. By this present work, we will complete the knowledge with data regarding the most prevalent ARB and ARGs present in these natural lakes. In this context, the aim of this study was to isolate and characterize the AR phenotypes (disk diffusion method) and ARGs (PCR method) in *Enterococcus* sp. and Gram-negative rods (mainly *Enterobacterales*) strains isolated in 2016 from Romanian aquatic fishery lowland salted lakes included in Natura 2000 Network [28], in the first attempt to evaluate their contribution to the AR aquatic reservoir.

## Results

The isolated strains were represented by 20 Gram positive cocci and 24 Gram negative rods which have been identified using the MALDI TOF MS, as: *Enterococcus faecium* (*n* = 8), *E. faecalis* (*n* = 7), *E. mundtii* (*n* = *2), E. casseliflavus* (*n* = 3), *Escherichia coli* (*n* = 9*), Enterobacter cloacae* (n = *2)* and one strain of each of the following species: *Klebsiella pneumoniae, K. oxytoca, E. kobei, E. ludwigii, E. cowanii, Escherichia hermannii, Serratia marcescens, S. rubidaea, Hafnia alvei, Pantoea ananatis, Raoultella ornithinolytica, Acinetobacter calcaoceticus*. The *Enterococcus* spp. strains have been isolated with different frequencies, depending on the isolation sources, i.e.: *E. faecalis* represented 20 % of the isolated strains and *E. faecium* only 5 % in Balta Albă lake (Table 1); *E. faecium* strains were isolated in proportion of 15 % from Câineni and Amara lakes, while from Jirlău lake *E. mundtii* and *E. casseliflavus* strains represented each 10 % of the isolated enterococci and *E. faecium* strains only 5 % (Table 1).

**Table 1:** The antibiotic resistance genes and antibiotic susceptibility profiles in the analyzed *Enterococcus* strains distributed by the isolation sources

Strain code	Species	Isolation source	ARGs			Antibiotic resistance profiles				
			Int	VanA	erm A	E	VA	TE	DXT	LZD
P1 1.1	*E. faecalis*	Balta Albă	X			X	X			
P1 1.2	*E. faecalis*	Balta Albă	X		X	X		X		
P1 1.3	*E. faecalis*	Balta Albă	X			X		X	X	
P1 1.4	*E. faecalis*	Balta Albă	X			X				
P1 1.5	*E. faecium*	Balta Albă								
P2 2.1	*E. mundtii*	Jirlău	X			X	X			
P2 2.2	*E. casseliflavus*	Jirlău	X							X
P2 2.3	*E. mundtii*	Jirlău	X			X	X			
P2 2.4	*E. casseliflavus*	Jirlău	X			X		X		
P2 2.5	*E. faecium*	Jirlău	X			X				
P3 3.1	*E. faecium*	Câineni	X			X				
P3 3.2	*E. faecium*	Câineni	X			X	X			
P3 3.3	*E. casseliflavus*	Câineni	X			X				
P3 3.4	*E. faecium*	Câineni	X			X	X			
P3 3.5	*E. faecalis*	Câineni	X	X	X	X	X			
P4 4.1	*E. faecium*	Amara	X		X					
P4 4.2	*E. faecium*	Amara	X				X			
P4 4.3	*E. faecium*	Amara	X			X				
P4 4.4	*E. faecalis*	Amara	X			X				
P4 4.5	*E. faecalis*	Amara	X							

**Fig. 1:**
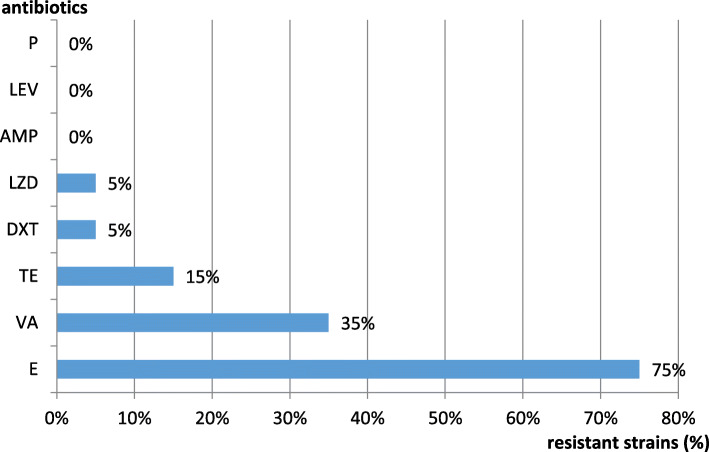
The antibiotic resistance profile of the *Enterococcus* spp. isolated strains revealed that 75 % of the strains were resistant to erythromycin, followed by vancomycin (35 %) and tetracycline (15 %), and 5 % of them to doxycycline and linezolid. Ampicillin and penicillin, the most active β-lactam antibiotics against enterococci were found to be 100 % efficient against isolated strains, as well as the tested fluoroquinolone (levofloxacin)

In our study, the disk diffusion susceptibility assay results revealed different resistance rates among the isolated *Enterococcus* spp. strains (Fig. 1).

The molecular support of the AR for all the strains isolated from fishery lakes was determined using simplex and multiplex PCR performed on genomic DNA in order to identify the genes encoding for resistance to β-lactams (*bla*_NMD_, *bla*_OXA−48_, *bla*_VIM−2_, *bla*_IMP_, *bla*_CTX−M_, *bla*_TEM_), PMQR markers (QnrA, QnrB, QnrS), sulfonamides (Sul1, Sul2), tetracyclines (TetA, TetB, TetC, TetD, TetM), aminoglycosides (aac3Ia), vancomycin (VanA, VanB, VanC), macrolides (ermA, ermB, ermC) and class 1 integrons (Int1, drfA1-aadA1). The molecular *screening* results were well correlated with the phenotypic assays, revealing the presence of VanA (*E. faecalis-*5 %) in vancomycin and of ermA genes (*E. faecalis* − 10 %, *E. faecium −* 5 %) in erythromycin resistant isolates (Table 1).

The distribution of the isolated GNR strains by the isolation sources revealed the following aspects: in Jirlău lake *E. coli* represented 16.66 % of the isolated strains followed by *E. cloacae* (8.33 %), *E. ludwigii* and *A. calcoaceticus* (4.16 % each) (Table 2). In the salty lake *E. coli* strains were predominant (16.66 %), followed by *R. ornithinolytica* and *E. hermannii* (4.16 % each). In Balta Albă lake there have been identified four different species and a high diversity have been observed also in Amara lake (Table 2).

**Table 2:** The ARGs and antibiotic susceptibility profiles of the analysed GNR distributed by the isolation sources

Strain code	Species	Isolation source	ARGs										Antibiotic resistance profiles							
			NDM	IMP	CTX-M	QnrS	Sul1	Sul2	TetC	TetA	aac3Ia	Int	KZ	CXM	CRO	CAZ	ATM	TIC	TE	K
P1 1.6	*S. marcescens*	Balta Albă			X		X					X	X	X				X		
P1 1.7	*K. pneumoniae*	Balta Albă									X	X		X				X		X
P1 1.8	*K. oxytoca*	Balta Albă			X		X											X		X
P1 1.9	*E. kobei*	Balta Albă		X			X					X						X		
P2 2.6	*A. calcaoceticus*	Jirlău			X		X					X	X	X	X	X	X	X		
P2 2.7	*E. ludwigii*	Jirlău					X						X					X		
P2 2.8	*E. cloacae*	Jirlău			X	X	X					X	X					X		
P2 2.9	*E. coli*	Jirlău			X		X					X	X	X				X		
P2 2.10	*E. coli*	Jirlău			X					X		X		X				X	X	X
P2 2.11	*E. coli*	Jirlău	X				X					X		X				X		X
P3 3.6	*E. coli*	Câineni			X				X			X		X				X	X	X
P3 3.7	*R. ornithinolytica*	Câineni					X					X		X				X		
P3 3.8	*E. coli*	Câineni								X		X		X				X	X	X
P3 3.9	*E. coli*	Câineni	X				X	X	X			X		X				X	X	X
P3 3.10	*E. coli*	Câineni					X			X		X						X		
P3 3.11	*E. hermannii*	Câineni					X					X						X		
P4 4.6	*K. oxytoca*	Amara			X		X		X			X	X					X	X	
P4 4.7	*E. cowanii*	Amara					X			X								X		
P4 4.8	*S. rubidaea*	Amara					X			X			X	X				X		X
P4 4.9	*E. coli*	Amara										X		X				X		
P4 4.10	*P. ananatis*	Amara					X					X	X	X				X		X
P4 4.11	*H. alvei*	Amara			X		X				X	X	X	X	X			X		
P 2.12	*E. cloacae*	Jirlău										X	X	X				X		X
P.2.13	*E. coli*	Jirlău										X						X		X

**Fig. 2:**
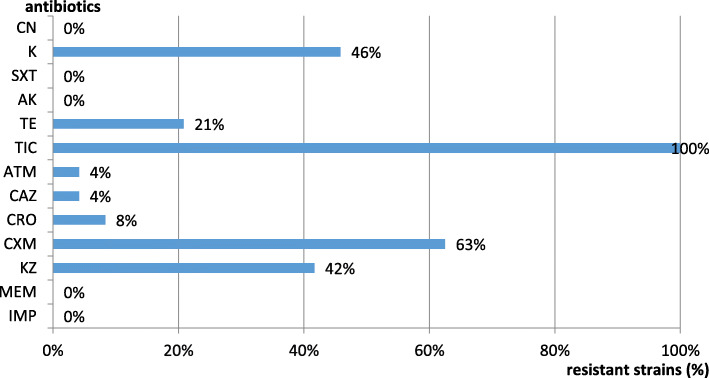
The antibiotic resistance profiles of the isolated GNRs. The analyzed GNR proved to be 100 % susceptible to aminoglycosides (excepting kanamycin), carbapenems, and trimethoprim/sulfamethoxazole. The GNR strains have shown 100 % resistance to ticarcilin, 21 % to tetracycline, 4 % to aztreonam and expressed different resistance levels to cefems (between 4 % and 63 %)

The analysed GNR strains expressed different resistance levels to β-lactam, aminoglycoside, tetracycline and trimethoprim/sulfamethoxazole antibiotics (Fig. 2).

Although some strains were resistant to antibiotics from different classes, they still remained sensitive to cephalosporins. This does not apply in case of the isolated *Acinetobacter calcoaceticus* strain, that proved to be resistant to all tested beta-lactams (data not shown).

The molecular study of the ARGs in the GNR strains revealed the presence of *bla*_CTX−M−_like (33 %), *bla*_NDM−_like and *bla*_IMP−_like (8.33 %) genes encoding for ESBLs and carbapenemases. The resistance to non-β-lactam antibiotics was ascertained to the following markers: PMQR (QnrS − 4.16 %); sulfonamides (Sul1–75 %, Sul2–4.16 %); aminoglycosides (aac3Ia − 4.16 %); tetracyclines (tetA – 25 %, tetC − 15 %) (Table 2).

## Discussion

This study investigated for the first time the AR features of cultivable microbiota of four lowland salted lakes of natural origin located in two counties from the southern region of Romania, namely Buzău and Brăila.

We observed an ubiquitary presence of *Enterococcus* spp. isolates, indicating the presence of chronic fecal pollution in all analyzed lakes (Table 1). Moreover, the *E. faecalis* strains isolated from Salty lake Câineni proved to be resistant to vancomycin (Van A) and erythromycin (ermA), exhibiting ARGs mostly found in clinical isolates. *Enterococci* are known to acquire relatively easy AR and to diseminate the ARGs to other species [29]. To date, nine distinct vancomycin resistance clusters have been described in enterococci (*vanA, vanB, vanC, vanD, vanE, vanG, vanL, vanM* and *vanN*), the *vanA* cluster being the most common mediator of vancomycin resistance in enterococci. Multidrug-resistant (MDR) and vancomycin-resistant *Enterococcus* (VRE) are now commonly isolated from clinical samples, sewage, aquatic enviroments, agricultural run-off and animal sources, which indicates their ability to enter the human food chain. VRE threatens to compromise the effective treatment of infections caused by these MDR bacteria, particularly in seriously ill patients who may need treatment with vancomycin where other antibiotics have failed [30]. The VRE conjugative transposons can transfer vancomycin resistance to *Staphylococcus aureus*, streptococci and lactobacilli. Historically, the vancomycin use was also coupled with the emergence and spread of methicillin-resistant *S. aureus* (MRSA) in the 1960s [31]. *E. faecalis* has been reported to transfer plasmids harbouring AR traits to other enterococci and to *Listeria monocytogenes* in wastewater treatment plants [32]. Also, *E. faecium* conjugative transposons can be transferred from animal to human microbiota.

The increased levels of erythromycin resistance observed in this study is also alarming, taking into account that macrolide-lincosamide-streptogramin (MLS) antibiotics constitute an alternative therapy for the treatment of insidious VRE infections [33]. Macrolide-resistant *Enterococcus* sp. strains have been often isolated in humans and animals [34, 35]. Two major resistance mechanisms that cause macrolide resistance in enterococci isolates are the target modification due to the ribosomal methylase encoded by erm genes (MLSB phenotype) and the expression of efflux pumps encoded by mef(A/E) and msr genes (M phenotype) [36]. Our isolates have shown the ermA genotype, affecting both macrolides and lincosamides.

Regarding the isolated GNR strains, our experimental data have shown that *E. coli* strains were isolated in a higher proportion in Jirlău and Câineni lakes compared to the other species (Table 2).

The emergence of GNR resistance in the aquatic environments can be associated with an increased risk to human and animal health [37–39]. Nowadays carbapenem-resistant *Enterobacteriaceae* are very difficult to treat especially in immunocompromised patients and these strains have also been reported in long-term care facilities [40]. Carbapenemases represent the most versatile family of β-lactamases, with a breadth of spectrum unrivalled by other β-lactam-hydrolysing enzymes. They have the ability to hydrolyze penicillins, cephalosporins, monobactams, and carbapenems. The most frequently reported carbapenemases in *Enterobacteriaceae* isolates in decreasing order are: NDM-1 (New Delhi metallo-β–lactamases) widely distributed among *Enterobacteriacae* and rapidly distributed around the world, OXA-48 identified in Turkey in 2001, OXA-181, a point mutant analogue of OXA-48, reported in India KPC; IMP-1 (imipenemase) first reported in *S. marcescens* in Japan in 1991; the most frequently carbapenemases in non-fermentative GNB are the VIM type (Verona imipenemase) firstly described in *Pseudomonas aeruginosa* isolates, which has been identified also in *Enterobacteriaceae* strains [41–43].

ESBLs are a group of plasmid-mediated enzymes that are posing a major therapeutic challenge today in the treatment of hospitalized and community patients. Most of ESBLs are members of TEM, CTX-M, OXA and SHV β-lactamase families [44]. CTX-M15 (the most commonly worldwide) was predominantly revealed in humans, while CTX-M1 in farming animals. The global spread of CTX-M type enzymes also associated with quinolones resistance has become the main concern nowdays. The trimetoprim-sulphamethoxazole resistance is most frequently encoded on plasmids coexpressing ESBLs [45, 46]. The ESBLs and carbapenemases have been reported as the major type of beta-lactams resistance mechanisms in both human and veterinary medicine among GNR. However, such resistance phenotypes were increasingly reported in the environment, which both receives and further disseminates MDR bacteria [47].

In the aquatic environments there have been demonstrated that efflux genes (tet A, B, C, D and E) are frequently detected in *Enterobacterales* species found in nature, humans, and animals [48]. The tet(A) gene is commonly associated with plasmids, and can be easily transferred between Gram negative bacteria [49].

In our study the integrase gene was found in more than 50 % of the investigated strains (58.33 %) exhibiting a MDR phenotype. Class I integrons are capable of transferring genes responsible for resistance to β-lactam, aminoglycoside, sulfonamide and quaternary ammonium salts [50]. Several studies have correlated high class 1 integron abundance in the environmnent to anthropogenic activity. In one study, concentrations were found to be higher in aquatic environments contaminated with metals and antibiotics compared to unexposed environments. Another study showed that class 1 integrons were more abundant in detergent and antibiotic-contaminated sewage sludge and pig slurry compared to unexposed agricultural soils. The capacities of mobile integrons to disseminate among bacteria, to confer adaptive advantages to continously changing environmental conditions, and to use the gene cassettes of the environmental metagenome make them likely facilitators of environmental AR dissemination [51].

Another study performed on 108 bacterial strains isolated from eels and aquaculture ponds, revealed the presence of some ARGs (*bla*TEM, tetC, sull, aadA, floR and qnrB) in a high percentage and also detected the class I and II integrons [52].

## Conclusions

The cultivable aquatic microbiota from fishery lakes is mainly represented by enterococci and *Enterobacterales* strains. The source of contamination of all these lakes is probably the neighboring urban sewage or factories. The GNR strains exhibited high levels of β-lactam resistance mediated by ESBL and metallo-β-lactamases, accompanied with a lower resistance to the majority of all other important classes of non- β-lactam antibiotics. The majority of *Enterococcus* spp. isolates were resistant to macrolides and vancomycin. The high level and diversity of resistance markers, correlated with a high frequency of class 1 integrons is suggesting that this environment could act as an important reservoir of ARGs with a great probability to be horizontally transmitted to other associated species in sediments microbiota, but also to animal and human pathogens raising the potential zoonotic risk for fish consumers. Thus, the findings of this study may assist in developing strategies to avoid the spread of ARB. The limitations of the article are represented by the small number of analyzed strains, the lack of details regarding the sources of pollution with ARB or other possible characteristics that might have explained the presence of resistant microorganisms. Further studies, some of them being already underway through ongoing projects will address these limitations in the future. For now, this article represents a red flag to warn about the resistant microorganisms found in Romanian natural salted lakes included in Natura 2000 network.

## Methods

### Sampling site and isolation of the strains

**Fig. 3:**
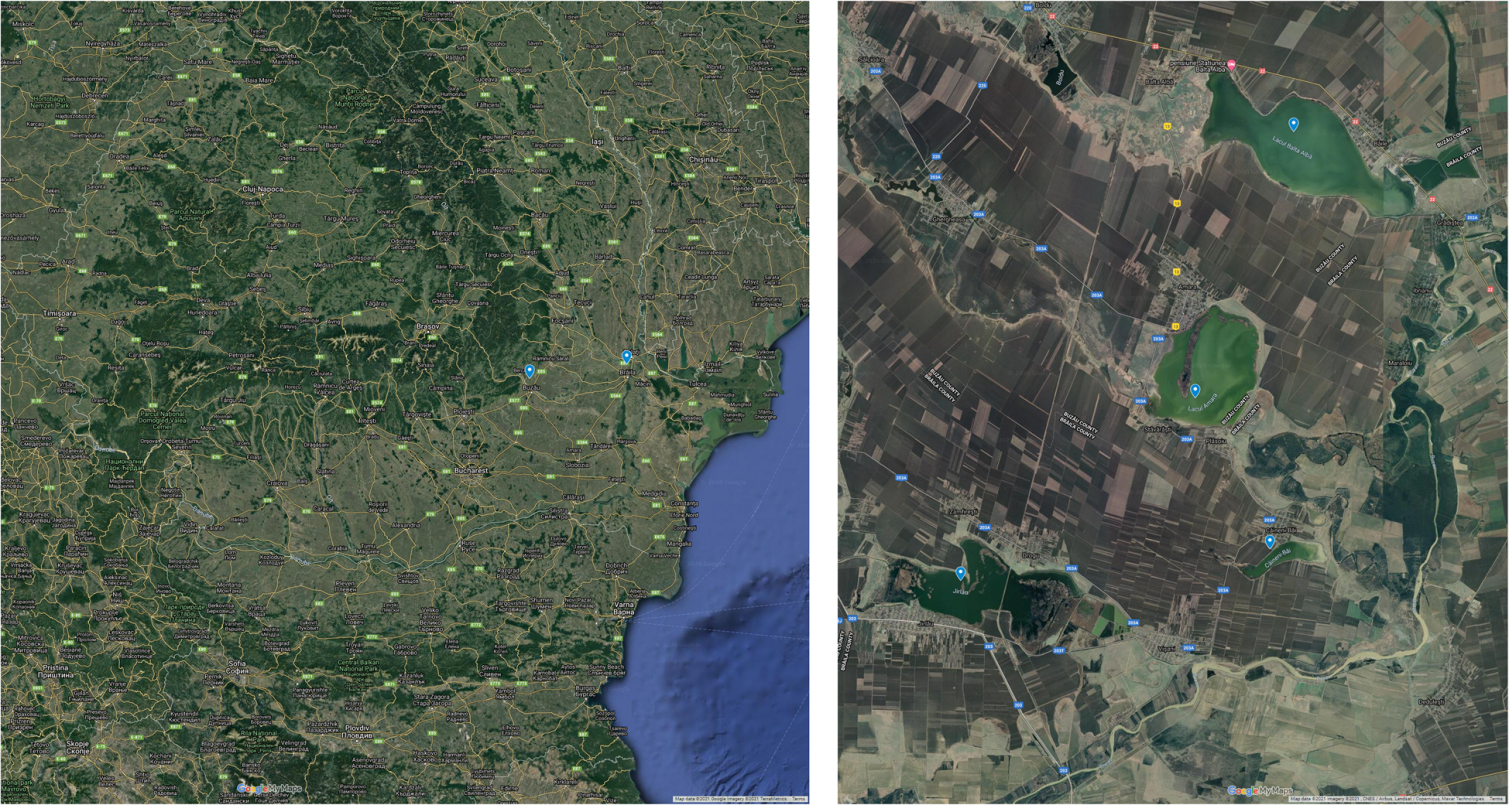
The geographic location of the investigated counties and the sampled four lakes from Buzău and Brăila counties (Map source: adapted after Google Maps)

The water samples were taken in sept. 2016 from four lowland salted lakes belonging to Natura 2000 network. The sampling points have been represented by four lakes circumscribed to a plain area of approx. 6500 ha of water from Buzău and Brăila counties, namely: ROSCI0005 Balta Albă-Amara-Lacul Sărat Câineni-Jirlău, ROSPA0004 Balta Albă-Amara-Jirlău and the area of reservations 2.271-Balta Albă, 2.272-Balta Amara, 2.260-Lacul Jirlău (Fig. 3). The area is polluted in particular by the actions and activities of the residents such as: discharging into the lakes of the domestic residues, natural and artificial modifications of the water composition, changing the methods of cultivation of land from the traditional ones in intensive agriculture and the excessive use of fertilizers. The lakes on this site are populated with fish of different species (carp, flax, chickpea, saltpeter and slumber), which are fished and consumed, and can be carriers of ARGs. The strains were isolated from samples on a selective (EMB, McConkey) and differential culture media (blood agar) as described previously [8]. A total of 44 lowland salted lakes strains were recovered from the selective and differential culture media and identified using the MALDI-TOF-MS Bruker system: 9 from the Balta Albă lake, 11 from the Balta Amara lake, 13 from the Jirlău lake and 11 from the salty lake Câineni.

### Antimicrobial sensitivity test for identified strains

The antibiotic susceptibility profiles were checked using disk diffusion method, according to Clinical and Laboratory Standards Institute (CLSI 2016 and 2017) using standard discs (µg/disc) (bioMérieux) for *Enterococcus* spp: penicillin (P, 10), ampicillin (AMP, 10), vancomycin (VA, 30), tetracycline (TE, 30), erythromycin (E, 15), doxycylyne (DXT, 30), levofloxacin (LEV, 15), linezolid (LZD, 30) and for different groups of Gram-negative bacteria, i.e.: cefazolin (KZ, 30), ticarcillin-clavulanic acid (TIC, 75 + 10), aztreonam (ATM, 30), meropenem (MEM, 10), imipenem (IMP, 10), cefuroxime (CXM, 30), ceftriaxone (CRO, 30), ceftazidime (CAZ, 30), gentamicin (CN, 10), amikacin (AK, 30), kanamycin (K, 30), tetracycline (TE, 30), trimethoprim-sulfamethoxazole (SXT, 1.25 + 23.75). After 24 h of incubation at 37 °C, organisms were classified as sensitive (S), intermediate (I) or resistant (R) based on CLSI break points.

### PCR detection of the resistance genes

The genetic support of the resistance [(carbapenemases – NDM, OXA-48,VIM-2, IMP), (ESBLs – CTX-M, TEM, SHV), plasmid-mediated quinolone resistance (PMQR) markers (QnrA, QnrB, QnrS), aminoglycosides (aac3Ia), sulphonamides (Sul1, Sul2), tetracyclines (TetA, TetB, TetC, TetD, TetM), macrolides (ermA, ermB, ermC) and class 1 integrons (Int1, drfA1-aadA1) was investigated by simplex (*bla*_SHV_, *bla*_OXA−51_, *bla*_OXA−58_; tetM, aac3-a; QnrS) and multiplex PCR (*bla*_OXA−48_+*bla*_NDM_; *bla*_CTX−M_+*bla*_TEM_; *bla*_OXA−24_+*bla*_OXA−24_; *bla*_OXA143_+*bla*_OXA235_; *bla*_IMP_+*bla*_VIM−2_; QnrA + QnrB; tetA + tetB; tetC + tetD; Sul1 + Sul2) (see the sequences of the primers - Table 3), using a reaction mix of 20 µl (PCR Master Mix 2x, Thermo Scientific) containing 1 µl of bacterial DNA extracted using an adapted alkaline extraction method. In this purpose 1–5 colonies of each strain was resuspended in 20 µl NaOH (0.05 M) + SDS (0.25 %), the suspensions were heated at 94 °C for 15 minutes followed by the addition of 180 µl of TE 1x buffer solution and centrifugation at 13,000 rpm, 3 minutes. The supernatant was checked by electrophoretic migration in a 1 % agarose gel and 1, 5 %, 45 minutes at 90 V and stained with 3.5 µg/ml ethidium bromide.

**Table 3:** Primers sequences used in simplex and multiplex PCR assays for β-lactams, quinolones, and aminoglycosides and tetracycline resistance genes

The gene	Primer	Nucleotide sequence	Amplification size and Tm	References
*bla*_OXA−48_	OXA-F OXA-R	GCGTGGTTAAGGATGAACAC CATCAAGTTCAACCCAACCG	438 52 °C	[53]
*bla*_NDM_	NDM-F NDM-R	GGTTTGGCGATCTGGTTTTC CGGAATGGCTCATCACGATC	621 52 °C	
*bla*_TEM_	TEM-F TEM-R	ATGAGTTTTCAACATTTTCG TTACCAATGCTTAATCAG TG	861 59 °C	[54]
*bla*_SHV_	SHV-F SHV-R	GCCCTCACTCAAGGATGTAT TTAGCGTTGCCAGTGCTCGA	888 58 °C	[55]
*bla*_CTX−M_	CTX-M-F CTX-M-R	CGCTGTTGTTAGGAAGTGTG GGCTGGGTGAAGTAAGTGAC	730 59 °C	[56]
*bla*_OXA−23_	OXA-23-F OXA-23-R	ATGAGTTATCTATTTTTGTC TGTCAAGCTCTTAAATAATA	501 52 °C	[57]
*bla*_OXA−24_	OXA24/40-F OXA24/40-R	GCAGAAAGAAGTAAARCGGGT CCAACCWGTCAACCAACCTA	270 52 °C	[58]
*bla*_OXA−51_	OXA-51-F OXA-51-R	TAATGCTTTGATCGGCCTTG TGGATTGCACTTCATCTTGG	353 52 °C	[57]
*bla*_OXA−58_	OXA-58-F OXA-58-R	AAGTATTGGGGCTTGTGCTG CCCCTCTGCGCTCTACATAC-	599 58 °C	[57]
*bla*_OXA−143_	OXA-143-F OXA-143-R	TGGATTGCACTTCATCTTGG TGGCACTTTCAGCAGTTCCT	180 58 °C	[59]
*bla*_OXA−235_	OXA-235-F OXA-235-R	TTGTTGCCTTTACTTAGTTGC CAAAATTTTAAGACGGATCG	700 58 °C	[59]
*bla*_IMP_	IMP-F IMP-R	GGAATAGAGTGGCTTAAYTCTC GGTTTAAYAAAACAACCACC	232 52 °C	[58]
bla_VIM−2_	VIM − 2-F VIM-2-R	GATGGTGTTTGGTCGCATA CGAATGCGCAGCACCAG	800 52 °C	[58]
QnrA	QnrAm-F QnrAm-R	AGA GGA TTT CTC ACG CCA GG TGC CAG GCA CAG ATC TTG AC	60 °C 580	This study
QnrB	QnrBm-F QnrBm-R	GGM ATH GAA ATT CGC CAC TG (M = A or C, H = A or C or T) GGM ATH GAA ATT CGC CAC TG (Y = C or T)	60 °C 264	
QnrS	QnrS-F QnrS-R	GCAAGTTCATTGAACAGGGT TCTAAACCGTCGAGTTCGGCG	428 60 °C	
aac3Ia	aac-3-IaF aac3IAR	ATGGGCATCATTCGCACA TCTCGGCTTGAACGAATTGT	59 °C	DQ370505
tetA	tetA-F tetA-R	GCRGCGATCTGGTTCACTCG AGTCGACAGYRGCGCCGGC	61 °C 164	[60]
tetB	tetB-F tetB-R	TACGTGAATTTATTGCTTCGG ATACAGCATCCAAAGCGCAC	61 °C 206	
tetC	tetC-F tetC-R	GCGGGATATCGTCCATTCCG GCGTAGAGGATCCACAGGACG	68 °C 207	
tetD	tetD-F tetD-R	GGAATATCTCCCGGAAGCGG CACATTGGACAGTGCCAGCAG	68 °C 187	
tetM	tetM-F tetM-R	ACAGAAAGCTTATTATATAAC TGGCGT GTCTATGATGTTCAC	55 °C 171	[61]
Sul1	Sul1-F Sul1-R	CGGCGTGGGCTACCTGAACG GCCGATCGCGTGAAGTTCCG	432 69 °C	[61]
Sul2	Sul2-F Sul2-R	GCGCTCAAG GCAGATGGCATT GCGTTTGATACCGGCACCCGT	293 69 °C	
Int	Int-F Int-R	ATGGCCGAGCAGATCCTGCACG GCCACTGCGCCGTTACCACCGC	899 60 °C	
dfrA1- aadA1	dfrA1- aadA1-F dfrA1- aadA1-R	AGCATTACCCAACCGAAAGT TGTCAGCAAGATAGCCAGAT	818 60 °C	

## Data Availability

All data analyzed or generated during this study are included in this published article and its Supporting Information files. Any additional information is available from the corresponding author on reasonable request.

## Abbreviations

AR: Antimicrobial resistance
JPIAMR: Joint Programme Initiative on Antimicrobial Resistance
ARGs: Antibiotic resistance genes
ARB: Antibiotic resistant bacteria
PMQR: Plasmid-Mediated Quinolone Resistance Determinants
GNR: Gram- negative rods
HGT: Horizontal Gene Transfer
MDR: Multidrug-resistant
VRE: Vancomycin-Resistant Enterococcus
MRSA: Methicillin-Resistant Staphylococcus aureus
MLS: Macrolide-Lincosamide-Streptogramin
NDM: New Delhi metallo- β-lactamases
IMP: Imipenemase
ESBL: Extended Spectrum β-lactamases
CLSI: Clinical and Laboratory Standards Institute
